# Case Report: Rehabilitation for Lower Extremity Pain Due to Venous Stasis in a Patient With Multisystem Inflammatory Syndrome in Children

**DOI:** 10.3389/fped.2021.810811

**Published:** 2022-01-06

**Authors:** Tokio Kinoshita, Yukihide Nishimura, Yasunori Umemoto, Yumi Koike, Ken Kouda, Takahiro Ogawa, Tomohiro Suenaga, Fumihiro Tajima

**Affiliations:** ^1^Department of Rehabilitation Medicine, Wakayama Medical University, Wakayama, Japan; ^2^Division of Rehabilitation, Wakayama Medical University Hospital, Wakayama, Japan; ^3^Department of Rehabilitation Medicine, Iwate Medical University, Shiwa-gun, Japan; ^4^Chuzan Hospital Clinical Education and Research Center, Okinawa, Japan; ^5^Department of Pediatrics, Wakayama Medical University, Wakayama, Japan

**Keywords:** case report, coronavirus disease, multisystem inflammatory syndrome in children, inflammation, rehabilitation

## Abstract

Recently, it was reported that children recovering from coronavirus disease (COVID-19) developed multisystem inflammatory syndrome in children (MIS-C), which causes severe inflammation in multiple organs of the body. Because MIS-C is a new disease, the pathophysiology and prognosis are unknown. Owing to a lack of studies on this subject, we herein provide information on rehabilitation for children with MIS-C. A 12-year-old male patient presented with systemic inflammatory symptoms after approximately 2 months since recovery from COVID-19. He was treated with cyclosporine and steroid pulse therapy after admission to our hospital. His general condition improved significantly within approximately 1 week. Thereafter, his lower legs turned dark purple and he experienced intense pain whenever the lower limbs hung below the heart, such as in the sitting position. The patient was referred to the rehabilitation department, as he had difficulties during standing and walking. Because the symptoms improved with elevation of the lower extremities, we considered that the pain was related to venous stasis. The pain reduced when an elastic bandage was applied for the prevention of venous stasis; therefore, exercise therapy was implemented while the patient wore the elastic bandage. The patient's lower extremity symptoms improved in 10 days. He was discharged after 16 days and could independently perform activities of daily living (ADL). The mechanism underlying the patient's pain could not be determined; however, rehabilitation was effective when combined with compression therapy using an elastic bandage.

## Introduction

The coronavirus disease (COVID-19) pandemic has devastated the world. Most children and adolescents infected with severe acute respiratory syndrome coronavirus 2 (SARS-CoV-2) often develop mild symptoms that do not require medical intervention (1, 2). However, in early May 2020, several European countries reported the outbreak of a condition in children that caused severe multiorgan inflammation, with features similar to those of Kawasaki disease shock syndrome and toxic shock syndrome, which was possibly related to SARS-CoV-2 infection (3–7). Health organizations (such as the World Health Organization and Centers for Disease Control and Prevention) referred to this syndrome as multisystem inflammatory syndrome in children (MIS-C) (8, 9). According to a preliminary definition, MIS-C is a hyperinflammatory syndrome that affects multiple organs, and its clinical features often overlap with those of Kawasaki disease. A systematic review conducted by Hoste et al. showed that patients with MIS-C had increased body temperature (99.4%); gastrointestinal (85.6%), cardiovascular (79.3%), shock (56.3%), and respiratory (50.3%) symptoms; and elevated levels of inflammatory biomarkers. Furthermore, most (73.3%) patients required intensive care, 3.8% of whom reportedly required extracorporeal membrane oxygenation (10). However, no study has reported the rehabilitation procedure for children with MIS-C.

Herein, we present a case of suspected MIS-C, wherein the patient underwent rehabilitation, which contributed to the improvement in his activities of daily living (ADL). The patient was admitted to our hospital in early March 2021. His symptoms were relieved with cyclosporine and steroid pulse therapy; however, during the disease course, he complained of pain when his upper and lower limbs were lowered below the position of the heart, and he could not accomplish the ADL.

## Case Report

**Figure 1:**
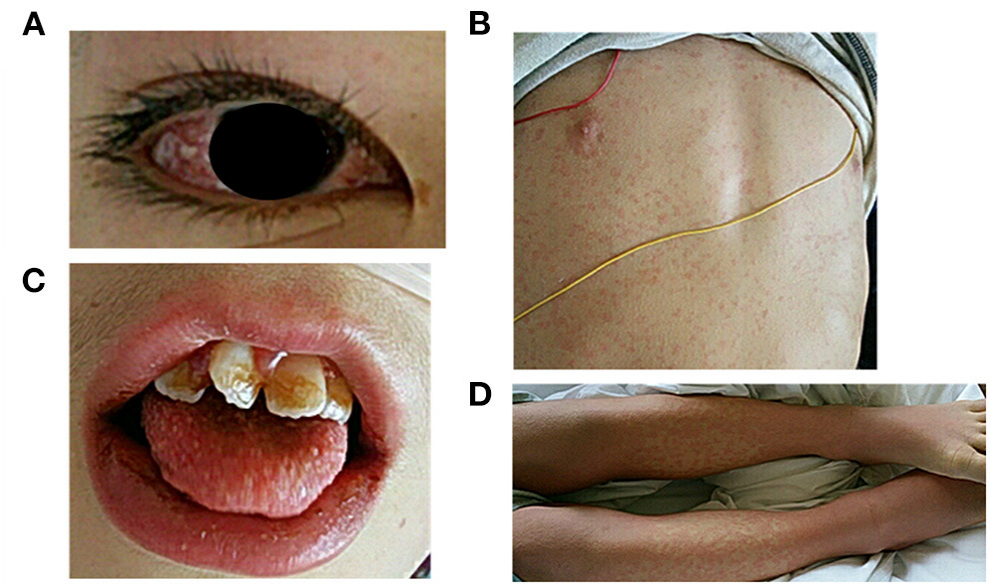
Visual examination performed at the time of admission to our hospital. **(A)** conjunctival injection, **(B)** skin rash, **(C)** erythema of the lips and strawberry tongue, **(D)** erythema and edema of the extremities.

A 12-year-old male patient (height and weight: 151 cm and 38.3 kg, respectively) was admitted to our hospital in January 2021 with a definitive diagnosis of COVID-19 (a positive result in polymerase chain reaction–based method) and was discharged after 1 week of complete recovery. At this time, the patient's symptoms of COVID-19 were mild, and the patient recovered with acetaminophen medication for fever. In addition, the patient had no notable complications thus far. In early March, he experienced abdominal pain and headache, and 3 days later, he developed fever (body temperature of 40°C); therefore, he visited a regional hospital and was admitted. Abdominal distention, generalized rash, and hyperemia of the conjunctiva appeared gradually in addition to high fever. The pediatrician made a suspected diagnosis of MIS-C because the symptoms resembled those of Kawasaki disease and speculated that the case was triggered by COVID-19 and consequently administered γ-globulins 85 g/day by intravenous drip to control the inflammation. After 4 days of hospitalization, his symptoms did not improve, even after γ-globulin administration, and his respiratory function decreased due to pulmonary edema. Hence, he was transferred to the pediatric department at our hospital, where he had a confirmed diagnosis of MIS-C (Figure 1). The patient was treated with cyclosporine (130 mg/day) and steroid pulse therapy (methylprednisolone sodium succinate 1,000 mg/day), after which his general condition gradually improved. On the 7^th^ day of admission to our hospital, the patient spent more time in bed, although he was able to sit and stand; hence, we encouraged him to walk within the ward to increase the level of activity. However, suddenly from this day, when the patient stood up or sat on the edge of the bed, his lower limbs turned dark purple, and he complained of numbness and pain. The pediatrician eliminated the possibility of an arterial lesion because there was no cold sensation in the lower extremities and the symptoms improved soon after elevation of the lower extremities. The patient underwent vascular Doppler sonography, which did not depict any significant thrombus. He presented with similar symptoms in the upper extremities.

On the 9^th^ day of hospitalization, the patient was unable to get out of bed. Therefore, he was referred to the rehabilitation department to alleviate the symptoms and start exercise therapy.

At the initiation of rehabilitation, his arterial blood pressure was 106/55 mmHg, pulse rate was 82 beats/min, oxygen saturation was 99%, and body temperature was 36.9°C. The patient's conjunctivae were not pale, and hyperemia was observed. There were no heart or lung murmurs. Echocardiography revealed an ejection fraction of 81.4%; normal right ventricular pressure; and extremely trivial regurgitation of the mitral, pulmonary, and tricuspid valves. The C-reactive protein level on the day of pain manifestation was 3.92 mg/dl, which dropped to 1.22 mg/dl at the start of rehabilitation. No exacerbation of inflammation was observed. The patient presented with clear consciousness (Glasgow Coma Scale: E4V5M6) (11, 12), and disorientation and communication problems were not observed. Manual muscle test (13) results showed that the muscle strength of both upper and lower limbs was level 4 (slightly lower than normal), and there was no obvious limitation in the range of motion of the joints. The patient had normal tactile, cold, and position sensation. The biceps and patellar tendon reflexes were in the normal range, and there was no increase in muscle tone. The patient was able to rise unaided from the supine position in bed to the edge sitting position. As soon as he sat on the edge of the bed, his lower limbs turned dark purple, and he complained of severe numbness and pain. The upper extremity symptoms that had been present on the other day disappeared, and the patient did not complain of pain during muscle strengthening exercises of the extremities in bed.

**Figure 2:**
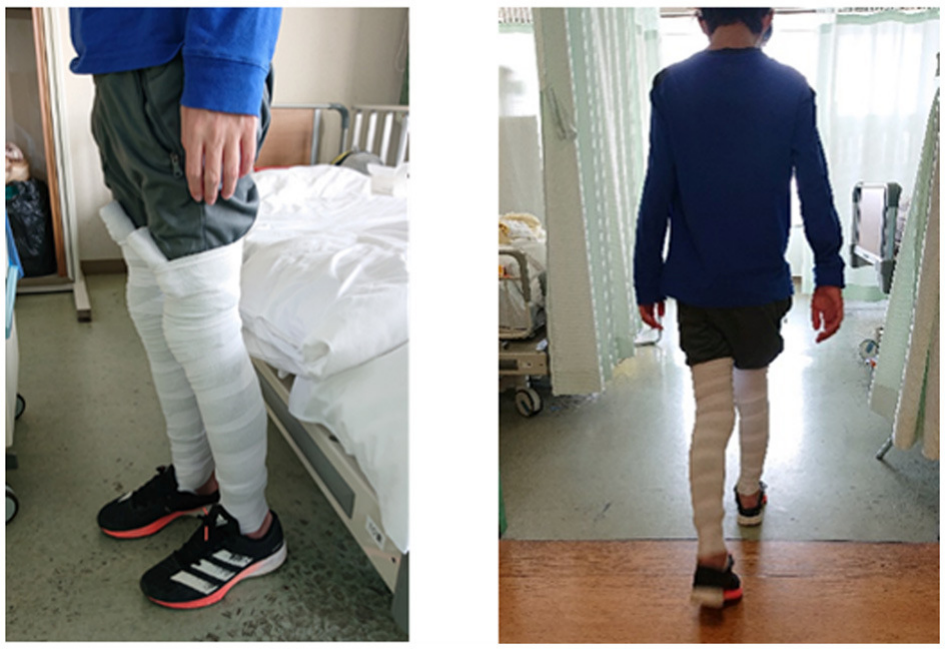
Images of the patient in the standing position and walking with elastic bandages on both lower limbs.

Because the patient's pain symptoms improved with lower extremity elevation, we hypothesized that venous stasis was a contributing factor. Therefore, exercise therapy was implemented, with the patient in the standing position and an elastic bandage wrapped only on the lower left extremity to reduce venous stasis. The patient then complained of pain only in the right lower extremity, indicating that reducing venous stasis would contribute to pain relief. Subsequently, elastic bandages were wrapped around both lower limbs, and the patient could stand and walk indoors (Figure 2). Although the patient complained of pain, he was able to walk for several meters. He was motivated to continue on the 2^nd^ day of rehabilitation because he was able to walk the previous day. He could walk indoors when aided by a nurse. The walking distance was extended during rehabilitation. On the 6^th^ day of rehabilitation, he was able to walk a short distance without wearing an elastic bandage. On the 7^th^ day, he was able to walk outside the ward (approximately 100 m) under supervision. An ergometer for the lower limbs was added to the rehabilitation routine to increase the exercise duration. On the 10^th^ day of rehabilitation, the patient no longer complained of pain in the lower limbs. On the 11^th^ day, he was able to bathe unaided, walk outdoors, and climb stairs. He was discharged on the 16^th^ day of rehabilitation (i.e., 23^rd^ day of admission) and could independently perform his ADLs.

Written informed consent was obtained from the patient and his family to publish any potentially identifiable images or data included in this article. This study conforms to all case report guidelines and reports the required information accordingly.

## Discussion

Several studies have reported rehabilitation for patients with COVID-19 pneumonia (14–18). However, to the best of our knowledge, there have been no reports of rehabilitation for patients with MIS-C. This case report is the first to describe rehabilitation for a patient with MIS-C-induced ADL deterioration.

The patient complained of pain when his lower limbs were lowered below the heart and was unable to get out of bed. Other diseases with similar symptoms, including chronic venous insufficiency and post-phlebitis syndrome, are caused by obstruction of venous return due to deep vein thrombosis, venous valve insufficiency, and decreased contractility of the muscles around the veins. Moreover, venous hypertension induced by fluid retention in the lower extremities due to right-sided heart failure may contribute toward the development of MIS-C. The symptoms include tenseness, heaviness, tingling, cramps, pain, and fatigue in the lower extremities, which exacerbate during standing or walking and improve with rest and elevation of the lower extremities. The treatment of chronic venous insufficiency and post-phlebitis syndrome entails elevation of the lower extremities, application of elastic bandages and stockings, and compression using pneumatic compression devices (19). Lauren et al. posited that MIS-C is an important factor in the pathobiology of both vascular inflammation and endothelial dysfunction (20). The patient did not have right-sided heart failure or venous obstruction, such as a thrombus, which would have interfered with venous return. We hypothesized that the inflammation had transiently spread to the veins of the patient, resulting in inadequate venous valve function and a tendency to develop stasis. There have been no reports of such symptoms occurring in children with MIS-C. The cause of symptom occurrence when the general condition was improving (rather than MIS-C onset) and the mechanism underlying pain generation were unknown. However, because the pain improved with elevation of the upper and lower limbs, we hypothesized that stasis in the veins was responsible for symptom occurrence. We successfully prevented venous stasis and reduced pain by applying an elastic bandage to the lower leg as symptomatic treatment. Compression therapy has been shown to reduce the amount of blood remaining in the veins of the lower leg and reduce the diameter of the veins, thereby improving the function of the venous valves and reducing venous reflux (21, 22). Moreover, studies have shown that the levels of pro-inflammatory cytokines produced by surrounding endothelial cells and macrophages due to venous stasis are reduced by compression (23, 24). It is possible that these mechanisms of action of compression therapy contributed to the reduction in pain. The patient's symptoms began to decrease after the initiation of rehabilitation. He was able to walk for a short distance without an elastic bandage by the 5^th^ day of rehabilitation, and the pain symptoms improved by the 9^th^ day. It is possible that the patient's symptoms could have been alleviated without rehabilitation. However, by adjusting the walking distance, endurance training load, and ADL training in a timely manner according to the pain level, the patient was able to return home early with ADL recovery.

Multisystem inflammatory syndrome in children is a new disease that was reported in April 2020 (3–7). Therefore, the pathogenesis and prognosis of this disease remain unclear. Furthermore, no study has reported rehabilitation for the same. Because this is a case report, it is difficult to generalize the progress of this patient to others with this condition. However, we believe that this study will provide valuable information regarding the rehabilitation of patients with MIS-C and their progress to remission. The patient has since returned to junior high school without any recurrence of symptoms, joined the basketball team, and is doing well in school without any problems.

## Conclusion

The pain experienced by the patient described in this study may not be a common symptom of MIS-C. However, for painful symptoms suspected to be related to venous stasis, rehabilitation was shown to be effective when used in conjunction with compression therapy with elastic bandages. This study suggests that rehabilitation should be performed for pain of unknown etiology, after carefully evaluating the physical findings of the patient.

## Data Availability Statement

The raw data supporting the conclusions of this article will be made available by the authors, without undue reservation.

## Ethics Statement

Ethical review and approval was not required for the study on human participants in accordance with the local legislation and institutional requirements. Written informed consent to participate in this study was provided by the participants' legal guardian/next of kin. Written informed consent was obtained from the minor(s)' legal guardian/next of kin for the publication of any potentially identifiable images or data included in this article.

## Author Contributions

TK and YN conceptualized and designed the study, drafted the initial manuscript, and reviewed and revised the manuscript. YU, TO, YK, KK, and TS designed the data collection instruments, collected data, carried out the initial analyses, and reviewed and revised the manuscript. FT designed the data collection instruments, coordinated and supervised data collection, and critically reviewed the manuscript. All authors approved the final manuscript as submitted and agree to be accountable for all aspects of the work.

## Conflict of Interest

The authors declare that the research was conducted in the absence of any commercial or financial relationships that could be construed as a potential conflict of interest.

## Publisher's Note

All claims expressed in this article are solely those of the authors and do not necessarily represent those of their affiliated organizations, or those of the publisher, the editors and the reviewers. Any product that may be evaluated in this article, or claim that may be made by its manufacturer, is not guaranteed or endorsed by the publisher.

## References

[1] Castagnoli R Votto M Licari A Brambilla I Bruno R Perlini S. Severe acute respiratory syndrome coronavirus 2 (SARS-CoV-2) infection in children and adolescents: a systematic review. JAMA Pediatr. (2020) 174:882–9. 10.1001/jamapediatrics.2020.1467 32320004

[2] Lu X Zhang L Du H Zhang J Li YY Qu J. SARS-CoV-2 infection in children. N Engl J Med. (2020) 382:1663–5. 10.1056/NEJMc2005073 32187458 PMC7121177

[3] Jones VG Mills M Suarez D Hogan CA Yeh D Segal JB. COVID-19 and Kawasaki disease: novel virus and novel case. Hosp Pediatr. (2020) 10:537–40. 10.1542/hpeds.2020-0123 32265235

[4] European Centre for Disease Prevention Control. Rapid Risk Assessment: Paediatric Inflammatory Multisystem Syndrome and SARS-CoV-2 Infection in Children (published May 15, 2020). Available online at: https://www.ecdc.europa.eu/en/publications-data/paediatric-inflammatory-multisystem-syndrome-and-sars-cov-2-rapid-risk-assessment (accessed October 10, 2021).

[5] Verdoni L Mazza A Gervasoni A Martelli L Ruggeri M Ciuffreda M. An outbreak of severe Kawasaki-like disease at the Italian epicentre of the SARS-CoV-2 epidemic: an observational cohort study. Lancet. (2020) 395:1771–8. 10.1016/S0140-6736(20)31103-X 32410760 PMC7220177

[6] Riphagen S Gomez X Gonzalez-Martinez C Wilkinson N Theocharis P. Hyperinflammatory shock in children during COVID-19 pandemic. Lancet. (2020) 395:1607–8. 10.1016/S0140-6736(20)31094-1 32386565 PMC7204765

[7] Royal College of Paediatrics and Child Health. Guidance: Paediatric Multisystem Inflammatory Syndrome Temporally Associated With COVID-19. (published 2020). Available online at: https://www.rcpch.ac.uk/sites/default/files/2020-05/COVID-19-Paediatric-multisystem-%20inflammatory%20syndrome-20200501.pdf (accessed October 10, 2021).

[8] World Health Organization. Multisystem Inflammatory Syndrome in Children and Adolescents Temporally Related to COVID-19. (published May 15, 2020). Available online at: https://www.who.int/news-room/commentaries/detail/multisystem-inflammatory-syndrome-in-children-and-adolescents-with-covid-19 (accessed October 10, 2021).

[9] Centers for Disease Control and Prevention. Emergency Preparedness and Response: Multisystem Inflammatory Syndrome in Children (MIS-C) Associated With Coronavirus Disease 2019 (COVID-19) (published July 16, 2020). Available online at: https://emergency.cdc.gov/coca/calls/2020/callinfo_071620.asp (accessed October 10, 2021).

[10] Hoste L Van Paemel R Haerynck F. Multisystem inflammatory syndrome in children related to COVID-19: a systematic review. Eur J Pediatr. (2021) 180:2019–34. 10.1007/s00431-021-03993-5 33599835 PMC7890544

[11] Teasdale G Murray G Parker L Jennett B. Adding up the Glasgow coma score. Acta Neurochir Suppl (Wien). (1979) 28:13–6. 10.1007/978-3-7091-4088-8_2 290137

[12] Teasdale G Maas A Lecky F Manley G Stocchetti N Murray G. The Glasgow Coma Scale at 40 years: standing the test of time. Lancet Neurol. (2014) 13:844–54. (Erratum in: *Lancet Neurol*. (2014) 13:863). 10.1016/S1474-4422(14)70120-6 25030516

[13] Kendall FP Kendall McCreary E Provance PG. Muscles—Testing and Function. 4th ed. Philadelphia, PA: Williams & Wilkins (1993). p. 179–90.

[14] Ricotti S Petrucci L Carenzio G Carlisi E Di Natali G De Silvestri A. Functional assessment and rehabilitation protocol in acute patients affected by SARS-CoV-2 infection hospitalized in the Intensive Care Unit and in the Medical Care Unit. Eur J Phys Rehabil Med. (2021). 10.23736/S1973-9087.21.06897-0. [Epub ahead of print]. 34605619 PMC9980591

[15] Patel N Steinberg C Patel R Chomali C Doulatani G Lindsay L. Description and functional outcomes of a novel interdisciplinary rehabilitation program for hospitalized patients with COVID-19. Am J Phys Med Rehabil. (2021) 100:1124–32. 10.1097/PHM.0000000000001897 34596096 PMC8594402

[16] Kinoshita T Kouda K Umemoto Y Yasuoka Y Minoshima Y Mikami Y. Case Report: a rehabilitation practice report during ICU management for a patient with multiple disabilities due to COVID-19 pneumonia and COPD. Front Med (Lausanne). (2021) 8:692898. 10.3389/fmed.2021.692898 34262919 PMC8274657

[17] Kinoshita T Umemoto Y Yasuoka Y Yoshikawa T Kouda K Hori S. Feasibility of sit training for patients with severe COVID-19 pneumonia during deep sedation: a case report. Medicine. (2021) 100:e26240. 10.1097/MD.0000000000026240 34087910 PMC8183700

[18] Leochico CFD Rey-Matias BMV Rey-Matias RR. Telerehabilitation perceptions and experiences of physiatrists in a lower-middle-income country during the COVID-19 pandemic. PM R. (2021). 10.1002/pmrj.12715. [Epub ahead of print]. 34585855 PMC8661588

[19] Mou M. Chronic venous insufficiency of the lower extremities and its treatment. Heart. (2016) 43:275–80 (in Japanese).

[20] Henderson LA Yeung RSM MIS-C early lessons from immune profiling. Nat Rev Rheumatol. (2021) 17:75–6. 10.1038/s41584-020-00566-y 33349661 PMC7750910

[21] Spence RK Cahall E. Inelastic versus elastic leg compression in chronic venous insufficiency: a comparison of limb size and venous hemodynamics. J Vasc Surg. (1996) 24:783–7. 10.1016/S0741-5214(96)70013-7 8918324

[22] Christopoulos DG Nicolaides AN Szendro G Irvine AT Bull ML Eastcott HH. Air-plethysmography and the effect of elastic compression on venous hemodynamics of the leg. J Vasc Surg. (1987) 5:148–59. 10.1016/0741-5214(87)90205-9 3795381

[23] Murphy MA Joyce WP Condron C Bouchier-Hayes DA. reduction in serum cytokine levels parallels healing of venous ulcers in patients undergoing compression therapy. Eur J Vasc Endovasc Surg. (2002) 23:349–52. 10.1053/ejvs.2002.1597 11991698

[24] Beidler SK Douillet CD Berndt DF Keagy BA Rich PB Marston WA. Inflammatory cytokine levels in chronic venous insufficiency ulcer tissue before and after compression therapy. J Vasc Surg. (2009) 49:1013–20. 10.1016/j.jvs.2008.11.049 19341889 PMC4710545

